# Exploring metazoan evolution through dynamic and holistic changes in protein families and domains

**DOI:** 10.1186/1471-2148-12-138

**Published:** 2012-08-03

**Authors:** Zhengyuan Wang, Dante Zarlenga, John Martin, Sahar Abubucker, Makedonka Mitreva

**Affiliations:** 1 The Genome Institute, Washington University School of Medicine, 4444 Forest Park Blvd, St. Louis, MO 63108, USA; 2 U.S. Department of Agriculture, Agricultural Research Service/ANRI, Animal Parasitic Diseases Lab, Beltsville, MD, 20705, USA; 3 Division of Infectious Diseases, Department of Medicine, Washington University School of Medicine, St. Louis, MO, 63108, USA; 4 Department of Genetics, Washington University School of Medicine, St. Louis, MO 63108, USA

**Keywords:** Proteins, Domains, Evolution, Metazoa, Vertebrates, Arthropods, Nematodes

## Abstract

**Background:**

Proteins convey the majority of biochemical and cellular activities in organisms. Over the course of evolution, proteins undergo normal sequence mutations as well as large scale mutations involving domain duplication and/or domain shuffling. These events result in the generation of new proteins and protein families. Processes that affect proteome evolution drive species diversity and adaptation. Herein, change over the course of metazoan evolution, as defined by birth/death and duplication/deletion events within protein families and domains, was examined using the proteomes of 9 metazoan and two outgroup species.

**Results:**

In studying members of the three major metazoan groups, the vertebrates, arthropods, and nematodes, we found that the number of protein families increased at the majority of lineages over the course of metazoan evolution where the magnitude of these increases was greatest at the lineages leading to mammals. In contrast, the number of protein domains decreased at most lineages and at all terminal lineages. This resulted in a weak correlation between protein family birth and domain birth; however, the correlation between domain birth and domain member duplication was quite strong. These data suggest that domain birth and protein family birth occur via different mechanisms, and that domain shuffling plays a role in the formation of protein families. The ratio of protein family birth to protein domain birth (domain shuffling index) suggests that shuffling had a more demonstrable effect on protein families in nematodes and arthropods than in vertebrates. Through the contrast of high and low domain shuffling indices at the lineages of *Trichinella spiralis* and *Gallus gallus*, we propose a link between protein redundancy and evolutionary changes controlled by domain shuffling; however, the speed of adaptation among the different lineages was relatively invariant. Evaluating the functions of protein families that appeared or disappeared at the last common ancestors (LCAs) of the three metazoan clades supports a correlation with organism adaptation. Furthermore, bursts of new protein families and domains in the LCAs of metazoans and vertebrates are consistent with whole genome duplications.

**Conclusion:**

Metazoan speciation and adaptation were explored by birth/death and duplication/deletion events among protein families and domains. Our results provide insights into protein evolution and its bearing on metazoan evolution.

## Background

Proteins convey the majority of biochemical and cellular activities in organisms. Their structural and functional units are defined as domains [1,2] where each protein may contain a single or multiple domains. Evolutionarily related proteins have been grouped into families. Member proteins from the same family usually share high functional and sequence similarity, and contain similar domain architectures [3,4]. Over the course of evolution, proteins undergo mutations, duplications, and domain shuffling [5], which can result in the generation of new proteins and protein families through natural selection. The interplay between the different protein evolutionary events creates complicated mechanisms that help govern speciation and adaptation of organisms [6]. It is believed that duplications can create functional redundancies and provide space for mutation and domain shuffling. Mutation and domain shuffling together with other genetic events can create functional variation and in some cases completely alter protein function. These changes subject proteins to natural selection and adaptation which in turn lead to the generation of new domains, proteins, protein families, and species. As such, analyzing these changes can greatly improve our understanding of protein evolution which in turn will enhance our perception of species diversity and adaptation. Such understanding can be of great economic importance. For example, identifying and characterizing protein families or domains unique to parasites, i.e. parasitic nematodes, can result in better disease treatment and control.

Protein evolution has been explored for decades. Indels and substitutions have been linked to protein structure and function [7,8]; gene duplication and protein family expansion have been correlated to organism adaptation [9-11], and; studies on protein domains have advanced our understanding of the protein repertoire [12,13]. Systematic studies of protein evolution, especially those that examine the relationships between domain evolution and protein family evolution have been limited by a dearth of sequence and functional data at the genomic level. However, recent and significant progress has been made in obtaining such data. Today, more than 5000 genomes of species encompassing a broad taxonomic distribution have been sequenced (http://www.ncbi.nlm.nih.gov/sites/entrez?db=genome), and their corresponding proteomes have been annotated. The culmination of these efforts is the emergence of databases consisting of well-defined protein domains such as Pfam [14], which define thousands of conserved protein domains with detailed information on sequence and function. These databases make possible methodical evaluations of protein evolution. Indeed, while our work was ongoing, Kawashima et al. [15] extracted important information on vertebrate adaptation from changes in domain architecture. Furthermore, Buljan et al. [16] found that changes in domain architecture are biased to the termini of proteins. These studies highlight the potential to glean important associations between domain evolution, protein family evolution, and species adaptation from systematic studies of protein and genomics databases.

The present investigation analyzed 9 metazoan proteomes covering the three major metazoan clades; vertebrates, arthropods, and nematodes, together with those of *Saccharomyces cerevisiae* and *Monosiga brevicollis* as outgroups. Using evolutionary and biostatistics methodologies, we evaluated deaths and births of protein families and domains, and duplications and deletions of protein family and domain members within the target species. Herein, we refer to the generation of new protein or domain families in a lineage as birth events and the disappearance of these families at a lineage as death events. To better illustrate the evolutionary dynamics, these events were summarized into four indices; change in protein family complexity, change in protein domain complexity, domain shuffling, and adaptation. We used these datasets to explore and provide new insights into metazoan adaptation, diversity and evolution.

## Results

### Protein family birth and death

Protein families were constructed from all sequences representing 11 eukaryotic taxa using Markov Clustering (MCL; [17]) where MCL clusters with multiple sequences were defined as protein families. In total, 17,752 families were identified from 151,044 proteins of the following 11 species; *Homo sapiens, Mus musculus, Gallus gallus, Drosophila melanogaster, Aedes aegypti, Bombyx mori, Caenorhabditis elegans, C. briggsae, Trichinella spiralis, Monosiga brevicollis* and *Saccharomyces cerevisiae* ( Additional file 1 and Additional file 2). These protein families have different taxonomic distributions with the majority of them aligning with specific clades of nematodes, arthropods, or vertebrates (Table 1 and Additional file 3). There are only 810 protein families having members present in all 11 taxa (hereafter referred as universal families). Nematodes have the highest number of specific families (6,613) among which, 1,087 are specific to *T. spiralis*. This is the highest number of families unique to a single species. In contrast, the arthropod lineage has the least number of specific families (2,045), and *G. gallus* has only 60 species-unique families (Table 2). The lineages leading to the Last Common Ancestor (LCA) of human and mouse (mammals) tend to have higher numbers of new family births, but it is the LCA of *C. elegans* and *C. briggsae* that has the highest number of family births. If normalized to branch lengths, family births in the LCA of mammals are the highest followed by births in the LCA of *C. elegans* and *C. briggsae*. *Trichinella spiralis* and *G. gallus* have twice as many family deaths as their neighboring taxa. After diverging, 932 families disappeared in *T. spiralis* compared with less than 460 in *C. elegans* or *C. briggsae*, 487 families in *G. gallus*, and less than 200 in *H. sapiens* or *M. musculus*. Among all the organisms examined, the lineage leading to *T. spiralis* exhibited the most family deaths. Overall, the numbers of family births are higher than family deaths and vary more than deaths over the lineages examined.

**Table 1:** Classification of protein families and domains

	**Total**	**Universal**	**Lineage Specific**		**Others**
			**Specific to species**	**Shared by species**	
Family	17,752	810	3,620	9,145	4,177
Domain	5,106	1,172	274	633	3,027

**Table 2:** **Birth and death evolutionary events**^**a**^

	**Branch**	**Family**		**Domain**		**Universal Fam.**		**Universal Dom.**	
**Lineage**^**b**^	**Length**	**Birth**	**Death**	**Birth**	**Death**	**Dupl**^**c**^	**Del**^**d**^	**Dupl**^**c**^	**Del**^**d**^
Hsa	0.05	177	129	39	42	251	706	950	4208
Mmu	0.06	106	96	27	48	245	668	709	4165
((Tsp (Cbr Cel)) ((Bmo (Aae Dme)) (Gga (Hsa Mmu))))	0.16	1274	59	645	76	1789	369	7541	1760
(Tsp (Cbr Cel)	0.13	147	548	21	303	102	2816	284	11139
((Bmo (Aae Dme)) (Gga (Hsa Mmu)))	0.09	805	36	175	27	474	1576	2538	5220
(Cbr Cel)	0.55	4564	379	91	159	658	1060	2118	3508
Tsp	0.62	1087	932	15	614	436	1506	1000	5778
Cbr	0.09	520	81	24	102	145	299	781	1264
Cel	0.09	295	51	31	91	174	319	1100	1044
(Bmo (Aae Dme))	0.18	725	446	37	261	695	1593	2104	7310
(Gga (Hsa Mmu)	0.33	2113	255	348	89	1567	2126	8494	7548
(Aae Dme)	0.14	452	205	13	78	137	758	396	2583
Bmo	0.38	273	574	34	394	270	1675	615	6864
Aae	0.28	346	363	28	328	652	1040	1693	4114
Dme	0.33	249	357	52	116	368	961	999	3472
(Hsa Mmu)	0.09	1144	50	123	32	304	329	1132	1409
Gga	0.12	60	487	24	342	87	1346	262	7607

^a^ Death/Birth events were normalized to branch lengths, Dupl/Del were normalized to both the branch lengths and the total number of universal families/domains.

^b^ Species codes: Tsp: *T. spirali*s; Cbr: *C. briggasae*; Cel: *C. elegans*; Bmo: *B. mori*; Aae: *A. aegypti*; Dme: *D. melanogaster*; Gga: *G. gallus*; Mmu: *M. musculus*;

Hsa, *H. sapiens*; ^c^ Duplication; ^d^ Deletion.

### Duplication and deletion in universal protein families

We selected 804 universal protein families containing members present in all 11 taxa and investigated duplications and deletions among the members. Focusing on universal families helped minimize the effects of species adaptation and detect signals associated with genomic evolutionary events. Six universal families were excluded because large numbers of sequences (more than 1,000) in those families prohibited further multiple sequence alignment and tree building. Of those examined, 12,507 duplications and 22,954 deletions were inferred, averaging 16 duplications and 29 deletions per family. In the majority of lineages, deletions outnumbered duplications; however, at the LCA of nematodes, deletions were 28 times greater than duplications suggesting protein families became smaller (Table 2). It appears there were two rounds of duplication bursts, one in the LCA of metazoans with an average of 2.2 duplications per family, and one in the LCA of vertebrates, which averaged 1.95 duplications per family. All other branches shared less than one duplication per family on average. Despite the variation in deletion events over different lineages, the numbers of deletions from the LCA to each present taxon were less variable than duplications. Comparing the terminal lineages, *G. gallus* had the fewest duplication events.

### Domain birth and death

We successfully identified 5,106 domains from 123,084 proteins. Unlike protein families where less than 5 percent were universal (810 out of 17,752) and more than 20 percent were species specific, more than 20 percent of domains (1,172 out of 5,106) were universal and less than 6 percent were species specific (Table 1).

Birth/death events of the 5,106 identified domains were inferred in the same manner as protein family birth/death events (Table 2). Domains had fewer birth/death events than protein families. Consistent with that observed in protein families, there was a burst of domain births in the LCA of metazoans and this was 2 times greater than that found in the LCA of arthropods and vertebrates after normalizing by branch lengths. However, different lineages exhibited dramatic variations in the number of death/birth events. The lineages leading to humans exhibited the largest number of domain births and the smallest number of domain deaths. In contrast, the lineages leading to *T. spiralis* showed the smallest number of domain births and the largest number of domain deaths. After the split, 614 domains disappeared in *T. spiralis* while approximately 250 domains disappeared in *C. elegans* and *C. briggsae* Since *T. spiralis* is a nematode parasite and lateral gene transfer has been reported in parasitic nematodes [18], details of the 15 domains born in *T. spiralis* were examined. Interestingly, 13 out of 15 have been annotated as bacterial or viral protein domains ( Additional file 4).

### Domain duplications and deletions

Similar to family member duplications and deletions, domain duplications and deletions were analyzed for each phylogeny. For the purpose of comparability, only the 1,168 universal domains (domains present in all 11 species) were considered. In total, 49,958 duplications and 94,648 deletions were inferred for the universal domains; 5 domains were excluded because they have more than 1,000 members. As observed among universal protein family members, domain duplication and deletion varied substantially over the course of evolution, and sister lineages did not have similar numbers of duplications and deletions (Table 2). However, domain duplications and deletions were more frequent than protein member duplications and deletions, averaging 43 duplications and 81 deletions per domain over the course of evolution for the species examined starting with the LCA of metazoa.

### Correlation between protein domain evolution and protein family evolution

Pearson’s correlation coefficients were used to investigate the relationship between domain evolution and protein family evolution (Table 3). Coefficients between different events of the same target (i.e., between death and birth of protein families) were all negative, suggesting no significant correlation. As expected, duplications of universal domains positively correlated with duplications of universal protein families (r = 0.96, p = 4.50E-10), as did their deletions (r = 0.93, p = 8.35E-8). Protein domain deaths and protein family deaths also were positively correlated (r = 0.87, p = 4.54E-6). Unlike the close correlation between universal domain duplication and domain birth (r = 0.93, p = 8.35E-8), the correlation between protein family birth and duplication was minimal (r = 0.48, p = 0.051); protein family birth was more strongly related to domain birth and duplication. These results suggest that new protein family generation is involved in both domain duplication and new domain formation, and implicate a role for domain shuffling. It is interesting that there is no positive correlation (r = -0.15, p = 0.553) between member deletions of the universal families and new protein family birth. It could indicate that lost members of universal families might not be a major source for new protein family formation. Conversely, there was a duplication burst in the LCA of the metazoa coincident with a large number of new families born in that lineage.

**Table 3:** **Pearson's correlation coefficients (bold text) and their significance**^**a **^**(regular text) of different evolutionary events**

	**Family**		**Domain**		**Universal Families**		**Universal Domains**	
	**Birth**	**Death**	**Birth**	**Death**	**Duplication**	**Deletion**	**Duplication**	**Deletion**
**Family birth**		**-0.58**	**0.59**	**-0.57**	**0.48**	**-0.15**	**0.54**	**-0.24**
		0.016	0.012	0.017	0.050	0.553	0.024	0.355
**Family death**			**-0.40**	**0.87**	**-0.33**	**0.64**	**-0.41**	**0.72**
			0.108	4.5E-06	0.200	0.005	0.102	0.001
**Domain birth**				**-0.38**	**0.91**	**-0.01**	**0.93**	**-0.05**
				0.135	3.70E-07	0.969	8.3E-08	0.862
**Domain death**					**-0.37**	**0.45**	**-0.42**	**0.50**
					0.139	0.072	0.096	0.043
**Universal Families duplication**						**0.05**	**0.96**	**0.10**
						0.838	4.5027E-10	0.716
**Universal Families deletion**							**0.06**	**0.93**
							0.821	4.5E-08
**Universal Domains duplication**								**0.04**
								0.872
**Universal Domains deletion**								

^a^ Significance of Pearson's correlation coefficient was tested using t-distribution.

### Dynamic evolutionary changes over the phylogeny

**Figure 1:**
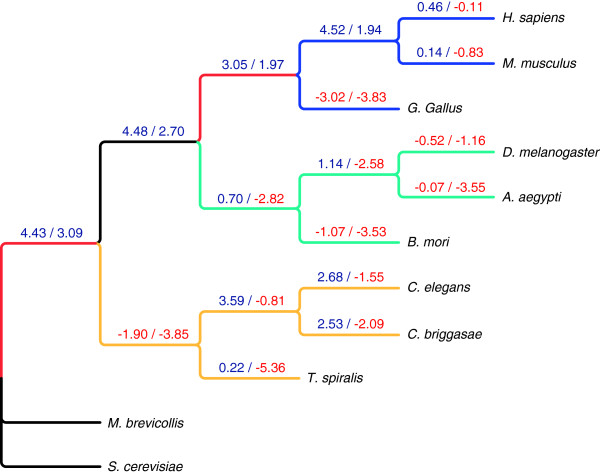
**Protein family and protein domain change indices. **At each lineage, the index for protein family change is followed by that of domain change (separated by back slash ‘/’). The index for protein family change was calculated using the log ratio of protein family birth and death events reconstructed from 17,752 homologous multimember families (151,044 proteins), thus representing how changes in protein families at any given lineage favor family gain or family loss. The index defining the change in protein domain complexity was calculated using the log ratio of protein domain birth and death events reconstructed from 123,084 proteins (5,106 domains). Analogous to protein family change, this represents how domain changes at any given lineage favor domain gain or loss.

Reconstructed birth/death events within protein families and domains provided opportunities to better understand evolution and adaptation. Over evolution, changes in the number of protein families differed from those of protein domains. As shown in Figure 1, most lineages (except for *G. gallus* and the LCA of nematodes) exhibited a gain in protein families, represented by the positive protein family indices, but the majority of the lineages also exhibited a loss in protein domains, represented by the negative domain indices. Nevertheless, the lineages leading to mammals exhibited domain gain (Figure 1). In contrast to the large gains in both protein families and domains of the LCA of vertebrates, the LCA of nematodes exhibited dramatic losses in both of these parameters. Interestingly, all other lineages of nematodes had gains in protein families. Compared to the lineages of nematodes and vertebrates (except for the LCA), arthropod lineages (except for the LCA) exhibited either less gain or more loss. In fact, all arthropod lineages exhibited a loss in protein domains. Among all organisms examined, however, the largest loss in domains was observed in *T. spiralis* (Figure 1). Overall the three metazoan clades showed different patterns of change. Consistent with the weak correlation between protein family birth and domain birth, little correlation in changes among protein families and domains was observed over the course of metazoan evolution.

**Figure 2:**
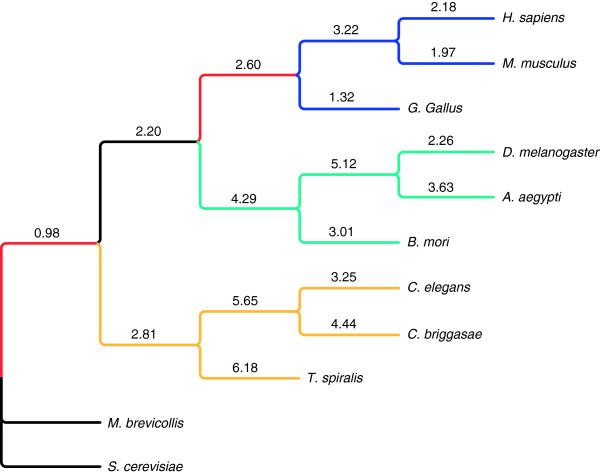
**Domain shuffling indices associated with the lineages over metazoan evolution. **The indices are the log ratio of protein family birth and protein domain birth events inferred in the corresponding lineage.

Given the lack of correlation between protein family and domain changes at all lineages, results suggest that domain shuffling played a large role in the formation of new families. To measure this effect we calculated the domain shuffling index i.e., the log ratio of protein family birth to protein domain birth, for each lineage (Figure 2). It is clear that the effects of domain shuffling in vertebrate lineages were less than those in arthropod and nematode lineages. This is in stark contrast to the strong increase in protein family complexity observed in vertebrate lineages. Meanwhile, domain shuffling appeared to have the strongest effects in the evolution of nematodes, where the terminal lineage of *T. spiralis* had the highest value (Figure 2). Consistent with the smallest number of duplications in protein families and domains, the terminal lineage of *G. gallus* exhibited the smallest domain shuffling index.

**Figure 3:**
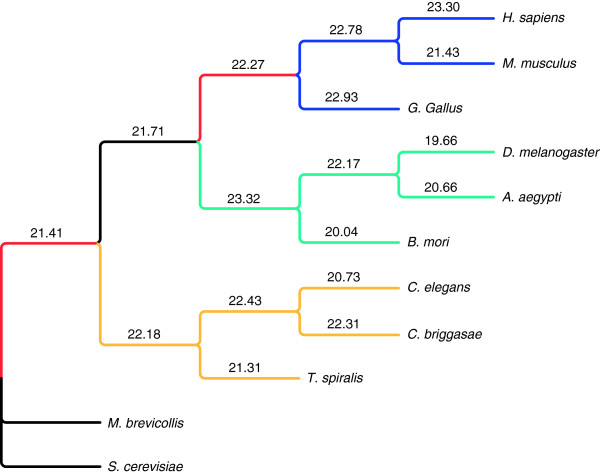
**Adaptation indices associated with metazoan lineages. **The indices were the summation of the logarithm of protein family birth events and death events, inferred at the corresponding lineages, normalized by the branch length of the lineage.

Complexity changes and domain shuffling indices did not inform us on temporal issues related to organism adaptation during evolution. To this end, we utilized the summation of the logarithm of protein family birth events and protein family death events normalized by lineage branch lengths as an adaptation index to define the speed of adaptation at the various lineages (Figure 3). Although the values in Figure 3 are additive and suggest a relatively constant but increasing adaptation index for all lineages, overall, this index did not exhibit significant differences among the lineages suggesting that adaptation has remained constant.

### Domain shuffling and protein family formation

**Figure 4:**
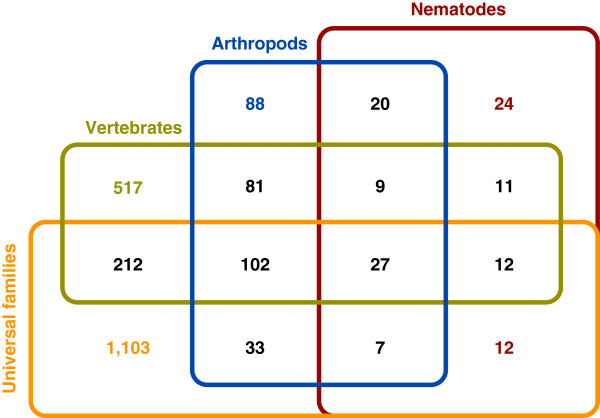
Distribution of protein domains among the protein families at the last common ancestor (LCA) of each of the three metazoan groups and the universal families.

**Figure 5:**
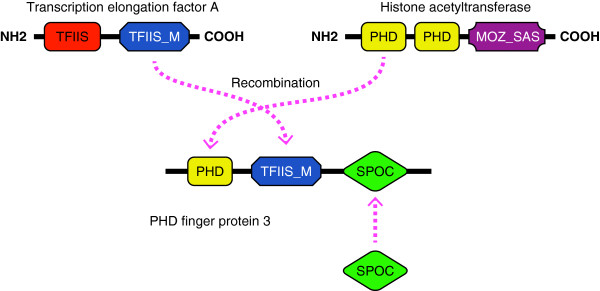
**A putative format for generating the vertebrate specific protein structure of PHD finger protein 3 (Cluster3894). **The domain structure of PHD finger protein 3 was formed through domain shuffling between universal families, transcription elongation factor A (Cluster1010) and histone acetyltransferase (Cluster330), followed by the addition of a new functional domain.

The above data suggest that domain shuffling has a strong impact on protein family complexity and organism adaptation. Consistent with this, a large number of domains of newly generated families were identified from existing domains. Figure 4 shows the numbers of domains in the protein families born to the LCA of the three metazoan groups, and how they overlap with each other and those of the universal families. For example, 120 domains were found within the 115 families born to the LCA of nematodes, 56 of which were found in the universal families. In addition, 63 of the 120 domains were found in the families born at the LCA of arthropods and 57 were present in families born at the LCA of vertebrates. These data indicate that in the process of generating new protein families, existing protein domains play a major role that involves domain shuffling. For example, the PHD finger protein 3 of vertebrates (Cluster3894) could have been generated by first shuffling between members of the ancient proteins transcription elongation factor A (Cluster1010) and histone acetyltransferase (Cluster330), followed by the addition of a new functional domain (Figure 5).

### Functional adaptation

The functions of families born at the LCAs of the three major clades and those born at the LCA of metazoans were investigated by biological process GO term enrichment/depletion. The GO terms enriched/depleted in these families closely align with adaptation of the species (Table 4). The most significant GO terms for the families born at the LCA of nematodes are G-protein coupled receptor protein signaling pathway (p = 3.54E-116), cell wall catabolic process (p = 7.49E-6), trehalose biosynthetic process (p = 2.37E-5), and cation transport (p = 2.98E-4). The most significant GO terms for families born at the LCA of arthropods are chitin metabolic process (p = 1.08E-22), sodium ion transport (p = 2.32E-18), response to stress (p = 2.02E-11), and sensory perception of smell (p = 4.74E-11). The top four enriched terms for the families at the LCA of vertebrates are G-protein-coupled receptor protein signaling pathway (p = 7.41E-155), immune response (p = 1.29E-39), regulation of cell growth (p = 2.57E-10), and cell communication (p = 4.19E-10). Upon a more broad examination of the data, the top four significantly enriched GO terms for the families born at the LCA of metazoans are regulation of DNA-dependent transcription (p = 1.29E-106), neurotransmitter transport (p = 7.59E-11), multicellular organismal development (p = 1.25E-9), and acyl-CoA metabolic process (p = 4.33E-8).

**Table 4:** **Enriched biological process GO**^**a **^**terms in protein families born at the LCA**^**b **^**of the three major metazoan groups and the LCA of metazoans**

**Groups**	**GO terms**	**Description**	**P-value**
Families born at the LCA of metazoans			
	GO:0006355	regulation of transcription, DNA-dependent	1.29E-106
	GO:0006836	neurotransmitter transport	7.59E-11
	GO:0007275	multicellular organismal development	1.25E-09
	GO:0006637	acyl-CoA metabolic process	4.33E-08
	GO:0007186	G-protein coupled receptor protein signaling pathway	1.35E-07
	GO:0007040	lysosome organization and biogenesis	4.78E-06
	GO:0007223	Wnt receptor signaling pathway, calcium modulating pathway	4.78E-06
	GO:0030704	vitelline membrane formation	6.47E-06
	GO:0006508	proteolysis	1.48E-05
	GO:0006094	gluconeogenesis	1.90E-05
	GO:0006665	sphingolipid metabolic process	3.24E-05
	GO:0007179	transforming growth factor beta receptor signaling pathway	3.24E-05
	GO:0006869	lipid transport	5.22E-05
	GO:0045449	regulation of transcription	9.33E-05
	GO:0006835	dicarboxylic acid transport	3.50E-04
	GO:0007600	sensory perception	3.50E-04
	GO:0045087	innate immune response	8.15E-04
	GO:0007026	negative regulation of microtubule depolymerization	9.14E-04
Families born at the LCA of nematodes			
	GO:0051085	chaperone cofactor-dependent protein folding	9.14E-04
			
	GO:0007186	G-protein coupled receptor protein signaling pathway	3.54E-116
	GO:0016998	cell wall catabolic process	7.49E-06
	GO:0005992	trehalose biosynthetic process	2.37E-05
	GO:0006812	cation transport	2.98E-04
Families born at the LCA of arthropods			
			
	GO:0006030	chitin metabolic process	1.08E-22
	GO:0006814	sodium ion transport	2.32E-18
	GO:0006950	response to stress	2.02E-11
	GO:0007608	sensory perception of smell	4.74E-11
Families born at the LCA of vertebrates			
			
	GO:0007186	G-protein coupled receptor protein signaling pathway	7.41E-155
	GO:0006955	immune response	1.29E-39
	GO:0001558	regulation of cell growth	2.57E-10
	GO:0007154	cell communication	4.19E-10
	GO:0050909	sensory perception of taste	4.88E-10
	GO:0045087	innate immune response	1.04E-09
	GO:0015671	oxygen transport	7.68E-09
	GO:0048468	cell development	1.03E-08
	GO:0006691	leukotriene metabolic process	7.08E-08
	GO:0042981	regulation of apoptosis	1.99E-07
	GO:0019882	antigen processing and presentation	3.04E-07
	GO:0009395	phospholipid catabolic process	3.60E-06
	GO:0006915	apoptosis	5.43E-06
	GO:0016049	cell growth	2.51E-05
	GO:0006486	protein amino acid glycosylation	3.09E-05
	GO:0006952	defense response	5.80E-05
	GO:0006071	glycerol metabolic process	8.96E-05
	GO:0009607	response to biotic stimulus	2.40E-04
	GO:0030178	negative regulation of Wnt receptor signaling pathway	2.54E-04
	GO:0043065	positive regulation of apoptosis	2.54E-04

^a^ GO, Gene Onthology; ^b^ LCA, Last Common Ancestor.

The functional association of family deaths at the LCAs of nematodes, arthropods, and vertebrates were also investigated through biological process GO term enrichment (Table 5). The top four enriched GO terms in family deaths at the LCA of nematodes are DNA catabolic process (p = 8.53E-8), DNA repair (p = 7.29E-5), regulation of Rho protein signal transduction (p = 2.1E-4), and porphyrin biosynthetic process (p = 2.65E-4); the top four enriched GO biological processes in families deaths at the LCA of arthropods are acyl-CoA metabolic process (p = 1.75E-18), vitelline membrane formation (p = 9.07E-18), lipid transport (GO:0006869, p = 2.08E-9), and sodium ion transport (p = 1.81E-8); and those in families deaths at the LCA of vertebrates are G-protein coupled receptor protein signaling pathway (p = 3.00E-12), intein-mediated protein splicing (p = 1.00E-7), cell communication (p = 3.28E-5), and chitin metabolic process (p = 9.21E-5).

**Table 5:** **Enriched biological process GO**^**a **^**terms in protein families died at the LCAs**^**b **^**of three major metazoan groups**

**Groups**	**GO terms**	**Description**	**P-value**
Families died at the LCA of arthropods			
	GO:0006637	acyl-CoA metabolic process	1.75E-18
	GO:0030704	vitelline membrane formation	9.07E-18
	GO:0006869	lipid transport	2.08E-09
	GO:0006814	sodium ion transport	1.81E-08
	GO:0045454	cell redox homeostasis	2.84E-08
	GO:0006555	methionine metabolic process	4.00E-04
	GO:0006952	defense response	5.87E-04
Families died at the LCA of nematodes			
	GO:0006308	DNA catabolic process	8.53E-08
	GO:0006281	DNA repair	7.29E-05
	GO:0035023	regulation of Rho protein signal transduction	2.10E-04
	GO:0006779	porphyrin biosynthetic process	2.65E-04
	GO:0006493	protein amino acid O-linked glycosylation	2.96E-04
	GO:0017000	antibiotic biosynthetic process	8.77E-04
Families died at the LCA of vertebrates			
	GO:0007186	G-protein coupled receptor protein signaling pathway	3.00E-12
	GO:0016539	intein-mediated protein splicing	1.00E-07
	GO:0007154	cell communication	3.28E-05
	GO:0006030	chitin metabolic process	9.21E-05
	GO:0006097	glyoxylate cycle	1.83E-04
	GO:0007275	multicellular organismal development	1.98E-04

^a^ GO, Gene Onthology; ^b^ LCA, Last Common Ancestor.

### Duplication of whole genome, protein families and domains

**Figure 6:**
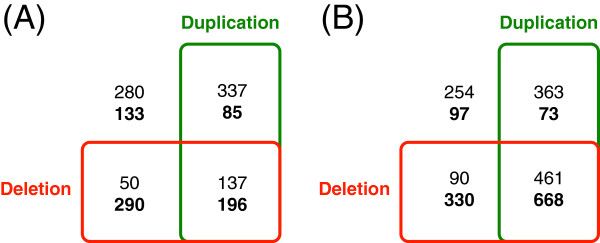
**Protein families (A) and protein domains (B) exhibiting duplication and/or deletion at the last common ancestor (LCA) of metazoans and at the LCA of vertebrates (bold). **The numbers of protein families and domains without any duplication or deletions are at the upper left corner.

As stated earlier, two family/domain duplication bursts were observed at the LCAs of metazoans and vertebrates. In order to evaluate the effects of whole genome duplication on these two bursts, the numbers of universal families/domains involved in duplications and/or deletions at these two LCAs were examined (Figure6). Results show that there are more families involved in duplications at the LCA of metazoans than at the LCA of vertebrates. Furthermore, when the numbers of families/domains involved in duplication only at these two LCAs were compared to those of families/domains involved in deletion only, the LCA of vertebrates had significantly lower values. The ratios of the vertebrate LCA were lower at 0.3 and 0.2 for family and domain, respectively, compared to the ratios of metazoan LCA at 6.7 and 4.0. These data strongly support whole genome duplication in the LCA of metazoans. Consistent with this, the universal families with only one member per species (113 families) had only 8 duplications at the LCA of vertebrates while the duplications at the LCA of metazoans numbered 61. In addition, the numbers of deletions and duplications were very similar at the LCA of vertebrates, but duplications were substantially greater than deletions at the LCA of metazoans for both universal protein families and universal domains (Figures 1 and 2). As such, the support for whole genome duplications at the LCA of metazoans is much stronger than support at the LCA of vertebrates.

## Discussion

This work provided a systematic analysis of both protein family evolution and domain evolution at the genomic level. Related evolutionary events were reconstructed and analyzed using proteomes from nine metazoan species via a variety of evolutionary and statistics programs. We included three well-annotated species, *H. sapiens*, *C. elegans*, and *D. melanogaster*, one for each major metazoan group, to reduce any bias from un-even annotation. We also included other less well characterized vertebrates, arthropods and nematodes for comparative purposes. To assure the reliability in reconstructing evolutionary events, yeast and choanoflagellate, the closest living relatives to metazoans, were included in the analysis as outgroups. In this way, we were able to relate both birth events and death events to species diversity and adaptation. This methodology allowed us to better explore any correlation between the evolution of protein families and protein domains, and reveal insights about species adaptation.

### Lineage specific protein family and domain evolution

Both birth/death and duplication/deletion of proteins and their domains vary substantially between lineages. We characterized these variations through four measurements; the log ratio of protein family birth and death (protein family change index), the log ratio of protein domain birth and death (protein domain change index), the ratio of protein family birth and protein domain birth (domain shuffling index), and the summation of logarithm family birth and death events normalized by branch length (adaptation index). Indices defining protein family and domain changes reflect family and domain gain or loss at different lineages, and represent changes in complexity of the organism’s proteome at each lineage. Domain shuffling index is a good indication for the effects of domain shuffling at any given lineage. The adaptation index illustrates how quickly adaptation occurred through protein family birth/death among the lineages during evolution. These measurements provided an interesting representation of metazoan, lineage-specific evolution.

Our study revealed a consistent increase in complexity during the evolution of vertebrate mammals from the perspective of protein families as illustrated by the positive change in the protein family and domain indices at the corresponding lineages. These data corroborate prior work on Pfam protein domains showing large increases in complexity among Metazoa where high rates of new domain formation and changes in domain architecture were observed [19]. The LCA of nematodes had a large reduction in complexity in both protein families and domains. However, unlike the lineages of arthropods, all three nematodes gained protein family complexity after splitting from their LCA (Figure 1). These data are consistent with the previously reported rapid generation of new protein families in vertebrates and nematodes [20].As reported, *G. gallus* exhibited a substantial reduction in protein family and domain complexities after it split from the LCA of vertebrates, and in general, the mean genome size is smaller in birds than in other tetrapods [21]. This occurs in concert with a reduction in ancestral protein-coding genes. Our results also showed that the terminal lineage of *G. gallus* had the largest protein family and domain losses and the smallest protein family and domain duplications. In addition, *G. gallus* exhibited the smallest domain shuffling effect. In fact, based on our results domain shuffling was less in vertebrates compared to arthropods and nematodes, with *G. gallus* having the lowest domain shuffling index (as shown in [22]) among vertebrates (1.32 vs. 1.97 for *M. musculus* and 2.18 for *H. sapiens*). It has been hypothesized that reduced genome size is the result of the evolution of flight and concomitant adaptation of birds to the high rate of oxidative metabolism needed for flying [21]. At this time, we cannot link the reduced genome size to reduced domain shuffling; however, less redundancy in the proteome in conjunction with strong selective pressures can effectively increase the deleterious effects of domain shuffling over time. This would result in an apparent reduction in domain shuffling in this lineage. Parasitism is clearly not analogous to flying; however, adapting to confined environmental niches marked by the evolution of parasitic nematodes or birds capable of flight is worth noting. *Trichinella spiralis* which unlike most parasitic nematodes has neither a free-living stage nor requires multiple hosts to complete its life cycle, is likely subject to fewer selective forces. Because of this, there are undoubtedly reduced requirements for the large repertoire of proteins demanded of free-living nematodes and those with more complicated life cycles, hence the remarkable reduction in complexity. In addition, the concept of a host-restricted animal, i.e., parasite, may result in the elimination of redundant protein families through the selection process [23]. For example, the nuclear receptor superfamily tends to vary largely among different species [24]. Free-living *Caenorhabditis* species possess hundreds of copies [10] whereas only 15 copies of this same receptor superfamily have been found in *T. spiralis*. This large disparity in copy number is accompanied by the loss of sub-family specific domains in *T. spiralis*. It has been hypothesized that the higher copy numbers are required in free-living nematodes for more efficient regulation of gene expression and for responding better and more quickly to environmental factors such as temperature, nutrient availability, metal ions or pH [10]. It follows therefore that fewer copies would be required in parasites such as *T. spiralis* that have adapted to a more predictable living environment. In contrast to *G. gallus*, the lineage of *T. spiralis* which has a very small genome relative to vertebrates, exhibited a strong domain shuffling effect. We believe that the high level of protein redundancy in *T. spiralis* resulting from host parasitism has made domain shuffling more tolerable during the evolutionary process.

Meanwhile, terminal lineages uniformly exhibited reductions in domain complexity while more than half of them showed increases in protein family complexity (Figure 1and 2). This suggests that new domains formed at a lower rate, and that domain loss outpaced domain gain at the terminal lineages. It is consistent with observations made by Lander et al. [25] who demonstrated that a near complete set of human gene domains is common to one or more lower eukaryotes as well. Even humans have gained only 39 domains while losing 42 domains (Table 1). Overall gains in protein family complexity were substantially larger than those of domain complexity at the different lineages examined. This is consistent with new protein families being generated by recruiting novel domains and by domain shuffling i.e. architecture rearrangement [26,27], which also includes domain (or gene) fusion and fission [28]. Because nematode lineages tended to lose domain complexity, the concomitant increase in protein family complexity suggests a strong contribution from domain shuffling over the course of their evolution (Figure 2). In contrast, mammals achieved protein family complexity by utilizing novel domains more so than other organisms. One possible source for these newly generated mammalian domains is genome duplication [29,30]. Our results also showed that LCA of Metazoa had low domain shuffling index. Our results also showed that LCA of Metazoa had low domain shuffling index. Although this node is quite distinct from the LCA of the Bilateria, this finding appears to conflict with prior observations indicating an increase in domain promiscuity, around the divergence of Bilateria [22]. However, our definition of domain shuffling is not synonymous with domain promiscuity, because domain shuffling as defined in our work takes into account the birth and death of both protein families and protein domains not taken into account by Cohen-Gihon et al [22]. For this reason, the addition of a large number of new domains as observed at the LCA of the Bilateria will automatically increase the domain promiscuity of ancient domains, calculated using the abundance of different domain architectures. Indeed, the LCA of Metazoa exhibited the largest number of domain births but a low shuffling index. Similarly, a reduction in the number of domains can decrease domain promiscuity; the likely reason why Cohen-Gihon et al [22] detected the smallest domain shuffling effect in *G. gallus.* We corroborated this finding where *G. gallus* exhibited a large number of domain deaths. Nevertheless, it is possible that our definition of domain shuffling index may underestimate the contribution of domain shuffling when there is a burst of domain births, like that observed at the LCA of Metazoa.

Aside from variations in complexities and the effects of domain shuffling, adaptation speed appears much less variable as represented by the more consistent adaptation indices in the different lineages (Figure 3). However, as SNP data has shown, there was a recent acceleration of adaptation in humans where demographic change, gene function, and gene-environment interactions could be key driving forces [31]. In our studies, the human lineages exhibited the highest adaptation index. It is highly likely that the same forces drove the fast protein family adaptation at the human lineage.

### Adaptation, and protein family and domain evolution

Our adaptation index derived from protein family birth and death did not reveal significant differences among the lineages studied. However, the differences in the numbers of protein and domain birth/death events and family member duplication and deletions in the lineages studied (Table 2) together with the variation of inferred changes in the indices defining protein family and domain complexities provide sufficient evidence of lineage specific adaptation. In turn, these lineage specific features and variations suggest a role for protein families and domains in species adaptation and diversity [32]. Adaptation related evolutionary variations have been reported multiple times. Taylor et al. [33] showed that the rate of protein duplication varied substantially among lineages. Hillier and coworkers [34] demonstrated that lineage-specific protein duplications and deletions were related to evolutionary change. Finally, Babushok et al. [13] reported that lineage-specific domain shuffling in different protein families promoted phenotypic complexity and species adaptation.

Functional enrichment of protein family births/deaths at the LCAs of the nematodes, arthropods, and vertebrates, and protein family births at the LCA of metazoans provide direct evidence for an association between species adaptation and protein evolution. Not surprising, protein families born at the LCA of metazoans were significantly enriched in functions related to regulation of transcription, multicellular organismal development, and signaling pathways, among others. These functional enrichments revealed the importance of the relevant families in the adaptation of metazoans while validating our family reconstruction. The most significant of enrichments, the regulation of transcription, is clearly reflective of the critical role this function plays in metazoan adaptation [35]. Similar to the results presented in our work, other studies have demonstrated that protein families encompassing signaling pathways and adhesion predated the origins of metazoans and were involved profoundly in metazoan adaptation [36-38], as were families involving neurotransmitter transporters [39]. New families related to the G-protein-coupled receptor protein signaling pathway are also enriched. Significantly more families related to this pathway were born at the LCA of nematodes and at the LCA of vertebrates. Interestingly, there was also a significant increase in the number of families of the same pathway that died at the LCA of vertebrates. These birth and death dynamics, especially those that occurred in the same lineage (i.e. the LCA of vertebrates), illustrate that G-protein-coupled receptor families as a whole played a significant role in metazoan speciation and adaptation [40,41]. For example, olfactory receptor proteins were found important for vertebrate diversity [42] as were protein families associated with cell communication. Given the complex configuration of tissues and organs in vertebrates, it is very likely that intercommunication between cell types is important in vertebrate evolution. Compared to signaling pathways, fewer enriched terms are associated with metabolism. This may relate to broad conservation in metabolic metabolisms in all metazoans. Therefore, further enrichment of the Acyl-CoA metabolic process in family deaths at the LCA of arthropods comes as little surprise. In support of this, the lack of acyl-CoA dehydrogenase homologues in arthropods has been previously reported [43]. We also noticed that GO term enrichment of families born at the LCA of vertebrates overlapped largely with the GO term enrichment of proteins under positive selection in mammals reported by Kosiol et al. [44]. This could reflect the role of positive selection on protein family dynamics.

Besides the signaling pathway involving the G-protein-coupled receptor, other families born at the LCA of nematodes with significant functional enrichment include the trehalose biosynthetic process, cation transport, and cell wall catabolic process. Trehalose may be used as a compatible solute to contend with osmotic stress or as an external carbon source [45-47]. In like manner, cation transport enrichment can also address osmotic stress [48]. The birth of families associated with cell wall catabolic processes may reflect the diversification in food resources connected with ancestral plant parasitic nematodes and subsequently coupled to free-living nematodes that followed. As such, additional analyses of enzymes involved in cell wall catabolism may reveal associations between parasitism and nematodes. Consistent with using externally-derived heme sources rather than synthesizing them de novo [49], porphyrin biosynthesis is among those protein families that died at the LCA of nematodes along with families associated with Rho protein signal transduction. Not much is known about nematode Rho signaling, but the absence of RhoBTB in *C. elegans* has been reported [50]. Additional families lost at the LCA of nematodes are those associated with the DNA catabolic process and DNA repair. Given the karyotype diversity, rapid genome changes, and chromatin diminution that occur in nematodes [51,52], it follows that the loss of protein families involved in the DNA catabolic process and in DNA repair could result in increased chromosome instability that can lead to these collective activities. To date there are no reports referencing evolutionary changes in DNA catabolic and/or repair pathways in nematodes; however, nematodes tend to have higher mutation rates than many organisms [53]. We are currently carrying out multiple nematode genome and transcriptome projects. Their completion will provide more data for better understanding the adaptation of nematodes. Expansion in some domains, especially those involved in signal transduction and DNA binding, were previously found to positively correlate with organism complexity [54]. However, our preliminary screen failed to confirm this, presumably because species with low numbers of domain copies and low numbers of cell types, such as protozoa and fungi were not included in this analysis (*S. cerevisiae* was included only as an outgroup). This could contribute significantly to the reported positive correlation.

### Whole genome duplication and burst of protein family/domain duplication

Despite the variation in protein family, and domain death/birth events, bursts of duplications were observed at the LCA of metazoans and at the LCA of vertebrates. The source for these bursts is not known, but whole genome duplication has been suggested for vertebrates and yeast [29,55]. The presence of duplication bursts confirm genome duplication within vertebrates, and the large number of deletions that accompanied the duplication bursts point to the difficulty in recovering the duplication history for vertebrates [30,56]. Meanwhile, our data strongly support genome duplication at the LCA of metazoans. There are more families involved in duplication than involved in deletion at the LCA of vertebrates, and a large fraction of these protein families are involved only in duplication. Though duplications and deletions inferred through tree reconciliation could suffer from the errors of tree estimation, systematic bias to a specific type of family is unlikely. In fact, other analyses involving separate protein families confirmed extensive duplications at the LCA of metazoans [57-59].

## Conclusions

Although the evolution of protein families and domains has been a research topic for some time, the current study is the first to closely investigate both duplication and birth/death rates for protein families and domains using a well-balanced and extensive data set. By reconstructing the evolution of protein families and domains over lineages that span the Metazoa which included all three major groups and multiple species within each group, for the first time we were able to quantify the relationship between protein family evolution and domain evolution, and examine the effects of domain shuffling in a lineage specific manner. By revealing the strong positive correlation between domain birth and duplication, we provided evidence for the evolutionary role of functional redundancy. By demonstrating a weak correlation between protein family birth and member duplication (in combination with the close correlation of the same events of domains), our analyses provided direct evidence for domain shuffling.

Additionally, we investigated not only new proteins that emerged (born) throughout evolution, but also proteins that disappeared (died) over this same period. This provided insights into understanding organismal adaptation, such as parasitism. To our knowledge, this is the first systematic study exploring adaptation through the death or disappearance of proteins. Finally, by examining both protein family and domain duplications, we provided strong evidence for whole genome duplication at the LCA of the Metazoa.

In conclusion, we studied metazoan evolution at a proteome level using a phylogenetic approach. Metazoan speciation and adaptation were explored by birth/death and duplication/deletion events among protein families and domains. The results characterized metazoan lineage-specific evolution related to protein families and domains. Despite the large variation, lineages leading to mammals exhibited consistent increases in protein family complexity during evolution. Results also illustrated that domain shuffling had a greater impact on protein family complexity in nematodes than in other metazoans, and that protein redundancy may be critical for evolutionary changes controlled by domain shuffling. By relating the evolutionary events to the functions of the proteins/domains involved, the results exposed the adaptive roles of these events. Overall, our study provides new insights into protein evolution associated with metazoan speciation.

## Methods

### Data collection

Whole proteome data from 9 metazoa were collected. The datasets were comprised of 3 species of vertebrates, 3 species of arthropods, and 3 species of nematodes. Data were downloaded as follows: *Homo sapiens, Mus musculus,* and *Gallus gallus* were from Biomart (http://www.biomart.org); *Drosophila melanogaster* and *Aedes. aegypti* were from Flybase; *Bombyx. mori* was from SilkDB[60]; *Caenorhabditis elegans* and *Caenorhabditis. briggsae* were from Wormbase[61]; and *Trichinella spiralis*[62]. The proteomes from the outgroups *S. cerevisiae* (yeast) and *M. brevicollis* (choanoflagelllate) were downloaded from Biomart (http://www.biomart.org) and JGI (http://genome.jgi-psf.org/), respectively. These proteomes were selected to keep the phylogenetic distances among the three species within each clade similar [63-66][64][65], [66] i.e., *H. sapiens* and *M. musculus* split about 100 million years ago (MYA) as did *C. elegans* and *C. briggsae*, and *G. gallus* split about 300 MYA from the ancestral vertebrates as did *T. spiralis* from ancestral nematodes. Isoforms of these downloaded sequences were examined against the coding genes, and only the longest ones were kept. The final dataset contained 22,997, 23,873, 16,736, 14,141, 15,419, 14,623, 20,188, 19,517, 16,124, 9,196, and 6,698 sequences from *H. sapiens, M. musculus, G. gallus, D. melanogaster, A. aegypti, B. mori, C. elegans, C. briggsae, T. spiralis, M. brevicollis* and *S. cerevisiae*, respectively.

### Protein family reconstruction

Protein sequences from the 11 species were searched (BlastP) against each other. Based on these results, we used MCL [17] to cluster the sequences and generate protein families according to Stein [65]. A value of 2.0 was chosen as the inflation factor for the MCL clustering because this was the optimum value to keep the homologous memberships between *D. melanogaster* and *S. cerevisiae* proteins identified by INPARANOID [67]. Protein families having members in all 11 species were defined as universal families.

### Domain identification

Each protein sequence was searched against the PFam domain profiles [14,68], using hmmpfam [69]. Significant matches were selected using the default cutoffs. The domain species and copies, and locations for each protein sequence were recorded. Based on this information, the sequences for every domain from the above proteins were extracted, and each domain was defined as a group. This process enabled an evaluation of domain evolution.

### Duplication and deletion detection

Duplications and deletions of protein sequences and domain sequences were identified using Urec [70]. First, the sequences for each family (or domain) were aligned using Muscle [71]. The distance matrices and reconstructed phylogenetic trees for each set of aligned sequences were computed using PRODIST and NEIGHBOR of Phylip [72], respectively. The reason for using NEIGHBOR instead of other likelihood-based programs was speed and because Urec considers only tree topology. We compared 20 random families using PROML and NEIGHBOR, and did not find any differences in the inferred tree topologies. We believe this topology consistency resulted from the large evolutionary distances among the organisms we analyzed. The reconstructed trees were reconciled with the species trees of the 11 taxa to infer the duplication and deletion events over their evolution using Urec. The relative rates of the corresponding events of each lineage were computed by normalizing the numbers of events using inferred branch lengths. These inferred branch lengths were derived from the multiple alignments of all universal families with single members per species using PROMLK of Phylip [72]. This permitted us to take advantage of molecular clocks among core proteins [73], and make the events comparable across different lineages.

### Protein family and domain death and birth

Using an approach similar to that of Hughes and Friedman [74], protein family death and birth were evaluated using DOLLOP [72] by treating each protein family as a character and its presence or absence as a discrete evolutionary state. A family member sequence from a species was assigned a value of 1 if it was found within that family (character), otherwise it was assigned a value of 0. DOLLOP reconstructed the ancestral states for all the characters (protein families) using a dollo parsimony algorithm [75]. Dollo parsimony is considered to overestimates the number of domains/proteins present in the most ancient nodes, however the use of Dollo parsimony does not always lead to overestimation in the most ancient nodes. This phenomenon is data dependent, and Dollo parsimony actually yielded lower ancestral intron densities than maximum likelihood (ML) based methods [76]. Because of this, we did not attempt to correct the bias. Dollo parsimony is based on simple assumptions, and is computationally cheap. In contrast, ML methods are usually computationally intensive, but more importantly require either an estimation of the rate of evolutionary change or force one to assume that the rate of change is constant. They can also produce significant bias when using an unrealistic rate of evolutionary change [77]. In our work like in many other evolutionary studies, the rate of change was not available. We expected great variation in evolutionary rates among different lineages (which our results confirmed), assuming a constant rate of change in all probability would have, severely violated the current analysis. For these reasons, we chose to use parsimony which is also the method of choice in evolutionary analyses when large and disparate datasets are involved. In addition, previous reports (e.g [78]) have also found that Dollo parsimony performed better than ML based methods in their gene content based tree reconstruction.

Protein family deaths and births were inferred by checking the states of these characters on each lineage of the 11 species tree. In a like manner, the death/birth events of each domain over its evolution were also inferred as were the unique domain losses of each species. Unique domain losses were defined as deaths of domains present in all other 10 species except the one indicated. The association of family member duplications/deletions, domain duplications/deletions, protein family deaths/births, and domain deaths/births were investigated using Pearson’s correlation coefficients. The significance of these correlation coefficients was tested using Student’s t-distribution.

### Indices defining protein family and domain change, adaptation, and domain shuffling

Organism complexity is closely related to the number of protein families and domains. In order to illustrate the changes of organism complexity over the course of evolution, we computed the log ratio of birth and death events of protein families and domains at different lineages, which we define as protein/domain change index. For example, if the number of birth events equals that of death events over a lineage, the index is 0, suggesting no complexity change over the evolution of this lineage. If the number of birth events is larger than death events, the index is larger than 0, suggesting the organism has gained complexity over the lineage.

The protein family and domain change indices reflect the changes of organism complexity, but does not reflect how quickly these changes occur. To assess the spread of these changes, we used the following equation:

(1) AI=log(B/l)+log(D/l)

where AI: Adaption Index; B: inferred birth events at the corresponding lineage; D: inferred death events at the corresponding lineage; l: the branch length of the corresponding lineage. If the branch length of a lineage is short and possesses a large number of birth/death events, then the adaptation index of that lineage is large, suggesting dramatic adaptation along that lineage. Only adaptation index of protein family was explored because of the limited number of domain birth events.

Protein families can be generated from new domains and/or domain shuffling. To illustrate the effect of domain shuffling in protein family evolution, we examined the log ratio of protein family birth to protein domain birth at different lineages which we defined as the domain shuffling index. Similar to the adaptation, index, the birth events were first normalized by the lineage branch lengths.

### Functional examination

GO term functional annotation of protein families was examined using Interproscan [79] based on *H. sapiens, D. melanogaster* or *C. elegans* proteomes. Significant enrichments of GO terms were computed based on hypergeometric distributions using FUNC [80] by comparing the numbers of a given GO term in the target group with the numbers in the background group. When testing a group of families, the GO terms identified by multiple members of the same family were counted only once. For example, when testing the GO term enrichment of nematode specific families, the GO terms identified by all *C. elegans* proteins were included and GO terms identified multiple times by different proteins from the same family were only counted once. When testing the probability of these data, refinement was performed by removing the GO terms identified as significant due to their derived terms. The false discovery rate (FDR) computed by FUNC was used to reduce false discovery. Therefore, unless specified otherwise, GO term enrichment was selected based on both p-value <0.001 (after refinement) and FDR <0.1.

## Abbreviations

FDR: False discovery rate; GO: Gene Ontology; LCA: Last common ancestor.

## Competing interests

The authors declare no competing financial interests.

## Author’s contributions

ZW, DZ and MM designed the study and wrote the manuscript. ZW, SA, and JM did the analysis. All authors read and approved the final manuscript

## Supplementary Material

Additional file 1 **Data summary of the species included in the analysis. **The data provided represent summary of the number of proteins and domains per species and proteins within protein families. Click here for file

Additional file 2 **IDs of protein families and their members. **The data provided represent the accession numbers of the proteins grouped in protein families. Click here for file

Additional file 3 **Groups of protein families with different taxonomic compositions. **The provided table summarizes the numbers of protein families and their taxonomic distribution. Click here for file

Additional file 4 ** *Trichinella spiralis* specific domains, their putative origin and function. **List of the identified domains in *T. spiralis* having highest sequence similarity with non-metazoan species, their putative function and species of origin. (XLS 25 kb) Click here for file
